# P-489. Clinical and Healthcare Burden of Infectious Mononucleosis in Children and in Adolescents/Adults

**DOI:** 10.1093/ofid/ofaf695.704

**Published:** 2026-01-11

**Authors:** Elizabeth Goodman, Halit Yapici, Weiqi Jiao, Leija Hu, Zhwiei Liu

**Affiliations:** Merck & Co., Inc., Boston, Massachusetts; Boston Strategic Partners Inc., Boston, Massachusetts; Boston Strategic Partners Inc., Boston, Massachusetts; Boston Strategic Partners Inc., Boston, Massachusetts; Merck & Co., Inc., Boston, Massachusetts

## Abstract

**Background:**

The need for an Epstein-Barr virus (EBV) vaccine is well recognized. EBV vaccine candidates have focused on prevention of infectious mononucleosis (IM), the most common clinical manifestation of primary EBV infection. Although most US children experience a primary EBV infection by age 10, IM is described as a disease of adolescents/young adults. Data on IM burden, including clinical characteristics and healthcare resource use, are scarce and no studies within the past decade describe IM burden in US children. This study describes clinical characteristics and health care resource use associated with IM in 2018 in the US and explores differences in IM burden between children less than 10 years of age and individuals aged 10 and older (adolescents/adults).

**Methods:**

TriNetX clinical data repository study of individuals in the with an ICD-10-CM code for IM or at least one positive EBV blood test (heterophile antibody and/or EBV serology) in 2018. IM cases were identified based on EBV serology results and/or presence of an ICD-10-CM code for IM. Sociodemographic and clinical variables and healthcare resource use were described and differences between children and adolescents/adults explored.

**Results:**

7443 cases of IM were identified, 10.9% of whom were children. Compared to adolescents/adults, children with IM were more likely to be male (52.5% vs 35.4%) and less likely to be non-Hispanic White (48.7% vs 73.5%) (p< 0.001). Blood testing was more common in children than adolescents/adults (40.3% vs 31.2%, p< 0.001). Lymphadenopathy, lymphocytosis, rash, airway obstruction and pneumonia were more prevalent in children (p< 0.001 for all), while hepatic involvement, fatigue, anxiety, and depression were more prevalent in adolescents/adults (p< 0.05 for all). Children were more likely to have ER visits and be hospitalized than adolescents/adults, who were more likely to be seen in ambulatory settings (p< 0.001).

**Conclusion:**

Clinical characteristics and healthcare resource use in children with IM are significant and, in some instances, greater than that seen in adolescents/adults with IM. The perception that primary EBV infection in childhood is asymptomatic or mild and that IM is a disease of adolescence and young adulthood may need to be reconsidered.

**Disclosures:**

Elizabeth Goodman, MD, MBA, Merck & Co., Inc.: Stocks/Bonds (Private Company) Halit Yapici, MD, MBA, MPH, Merck: Employee of BSP (supported by Merck for this research) Weiqi Jiao, ScM, Merck & Co., Inc.: Employee of Boston Strategic Partners (Supported by Merck for this research) Leija Hu, n/a, Merck: employee of BSP (supported by Merck for this research) Zhwiei Liu, PhD, Merck & Co., Inc.: Stocks/Bonds (Private Company)

